# Biomarkers of Parkinson’s Disease: From Basic Research to Clinical Practice

**DOI:** 10.14336/AD.2023.1005

**Published:** 2024-08-01

**Authors:** Zi-lu Ma, Zhang-li Wang, Fei-yue Zhang, Hong-xun Liu, Li-hong Mao, Lin Yuan

**Affiliations:** Laboratory of Research in Parkinson’s Disease and Related Disorders, Key Laboratory of Major Chronic Diseases of Nervous System of Liaoning Province, Health Sciences Institute, China Medical University, Shenyang, China.

**Keywords:** Parkinson’s disease, α-synuclein, biomarkers, diagnosis

## Abstract

Parkinson’s disease (PD) is a common neurodegenerative disease characterized pathologically by dopaminergic neuron loss and the formation of Lewy bodies, which are enriched with aggregated α-synuclein (α-syn). PD currently has no cure, but therapeutic strategies are available to alleviate symptoms. Early diagnosis can greatly improve therapeutic interventions, but the clinical diagnosis of PD remains challenging and depends mainly on clinical features and imaging tests. Efficient and specific biomarkers are crucial for the diagnosis, monitoring, and evaluation of PD. Here, we reviewed the biomarkers of PD in different tissues and biofluids, along with the current clinical biochemical detection methods. We found that the sensitivity and specificity of single biomarkers are limited, and selecting appropriate indicators for combined detection can improve the diagnostic accuracy of PD.

## 1. Introduction

Parkinson’s disease (PD) is the second most common neurodegenerative disease. It is characterized by the loss of dopaminergic (DAergic) neurons in the substantia nigra (SN) and the formation of intraneuronal protein inclusions known as Lewy bodies (LBs). The clinical features of PD comprise motor symptoms, such as dyskinesia and resting tremor, and non-motor symptoms, such as gastrointestinal tract dysfunction and autonomic nerve dysfunction [1]. The prevalence of PD is 1% after the age of 60 and 3% after the age of 80 [2]. However, PD has no effective cure, mainly because the precise mechanism underlying its pathogenesis is not fully understood, and no effective targets are available for early diagnosis and treatment.

PD diagnosis currently relies mainly on clinical characteristics and imaging tests, but clinical symptoms only appear after over 50%-70% of DAergic neurons have degenerated; therefore, accurate clinical diagnosis is late, eliminating the opportunity for early treatment and intervention [3]. As PD enters the intermediate and late stages, in which clinical symptoms are easy to diagnose, the effect of drug treatment is greatly reduced [4]. Early diagnosis would help identify PD patients at the prodromal or preclinical stages, enabling them to begin receiving neuroprotective treatments as soon as possible [5, 6]. Although prodromal symptoms may arise many years before PD motor symptoms appear, no sufficiently specific diagnostic markers have been identified for the development of an efficient method to diagnose PD in the early stage [7]. The discovery of specific biomarkers in the body fluids of patients with PD would facilitate the monitoring of PD progression, and physicians would not have to rely only on structural, pathological, and functional brain imaging measurements.

**Figure 1.**
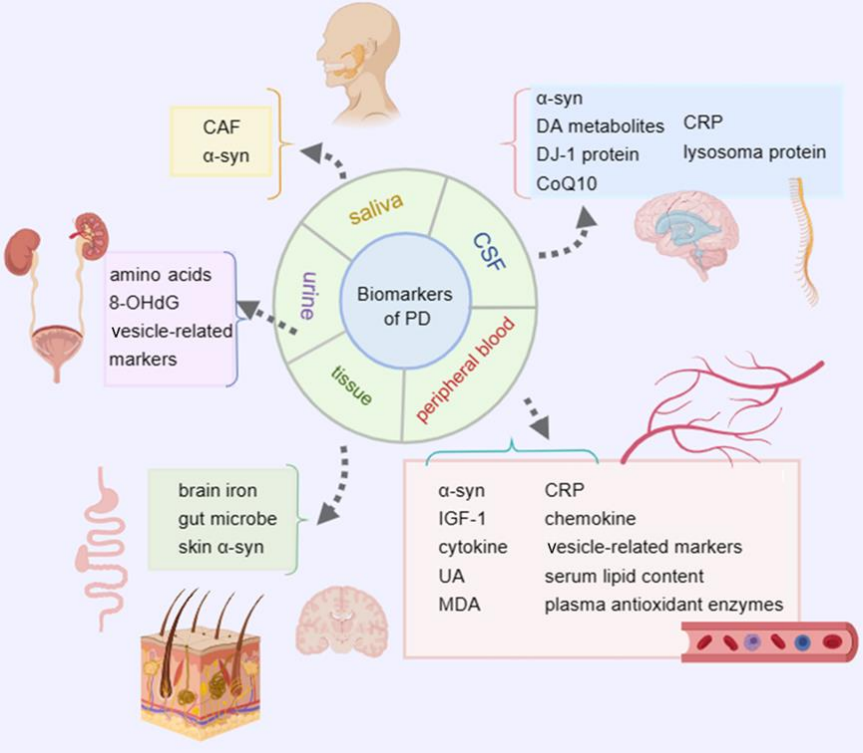
**Potential biomarkers for PD**. The potential biomarkers of PD from body fluid and tissues. Biomarkers in peripheral blood are labelled with a red background. Biomarkers in CSF are represented with a blue background. Yellow and purple backgrounds represent biomarkers in saliva and urine, respectively. Biomarkers in tissue are labelled with a green background. Some of the sensitive biomarkers are made into clinical kits and eventually used in clinical diagnosis.

Therefore, finding biomarkers with high sensitivity and specificity will be conducive to the early diagnosis, treatment, and monitoring of PD progression. Here, we focus on potential biomarkers of PD in body fluids (the cerebrospinal fluid (CSF), peripheral blood, saliva, and urine) and tissues (the brain, intestinal tract, and skin), as well as several clinical diagnostic assays and kits commonly used in clinical practice (Fig. 1).

## 2. Biomarkers in body fluids

### 2.1 Cerebrospinal fluid (CSF)

#### 2.1.1 α-Synuclein

α-Synuclein (α-Syn) is a soluble protein that is widely present in the central nervous system (CNS), but it also can be detected in plasma, saliva, and skin tissues [8, 9]. The results of most cross-sectional and longitudinal studies have shown that the level of total α-syn (t-α-syn) in the CSF of patients with PD is significantly lower than that in the CSF of healthy controls (HCs) [10-12] and that decline of t-α-syn is associated with cognitive decline [13]. However, the level of t-α-syn in the CSF is also reduced in other neurodegenerative diseases (NDDs), such as dementia with Lewy bodies (DLB) and multiple system atrophy (MSA); hence, the level of t-α-syn in the CSF cannot be used to distinguish PD from other NDDs [14]. α-Syn seed amplification (i.e., real-time quaking-induced conversion (RT-QuIC) and protein misfolding cyclic amplification (PMCA)) was recently shown to have a sensitivity and specificity of over 90% [15] and has been used to identify misfolded α-syn aggregates in the CSF and peripheral tissues. Additionally, these two techniques can separate PD from other NDDs according to variations in α-syn aggregates [16, 17]. Furthermore, studies on different variants of α-syn have found that the levels of oligomeric α-syn (o-α-syn) [18] nd phosphorylated α-syn (p-α-syn) at S129 (pS129 α-syn) are significantly increased in CSF of patients with PD [16, 19]. Because of the limitations of using a single marker, different forms of α-syn, such as the o-α-syn/t-α-syn ratio, can be utilized to improve the sensitivity and specificity of diagnosis [14].

#### 2.1.2 Dopamine Metabolites

Dopamine metabolites include 3,4-dihydroxypheny-lacetic acid (DOPAC) and homovanillic acid (HVA) [20, 21]. A previous study reported that concentrations of DOPAC and HVA were reduced in the CSF (with a 75% sensitivity and 100% specificity) of patients with early-stage PD and that those with lower levels of DOPAC were more likely to develop clinical signs of PD during follow-up [22-25]. The Deprenyl and Tocopherol Antioxidative Therapy of Parkinsonism (DATATOP) study reported high variability in the results of measuring dopamine metabolite levels in the CSF. Therefore, the feasibility of using dopamine metabolites to monitor PD progression has been questioned. However, in recent years, the absolute quantification of dopamine metabolites by liquid chromatography coupled with tandem mass spectrometry (LC-MS/MS) combined with clinical follow-up has enabled the reduction of the influence of the intrinsic variability in a proportion of a sample of patients, according to experimental results [22]. Therefore, DOPAC and HVA still have potential in PD diagnosis and efficacy assessment.

#### 2.1.3 DJ-1 protein

DJ-1 protein, also called oxidation-sensitive protein, is widely expressed in neurons and glial cells in the brain and exerts neuroprotective effects during neurodegeneration [26]. A previous study demonstrated that the level of DJ-1 in the CSF of patients with PD was reduced compared with that in the CSF of HCs [27]. DJ-1 has been linked to oxidative stress and mitochondrial homeostasis in PD cells; therefore, it has potential in cellular metabolic pathology [28, 29]. In addition, reduced DJ-1 expression levels were observed in the plasma of patients with prodromal PD, making it a candidate marker for early PD [30]. The therapeutic potential of DJ-1 to protect PD neuronal cells through signaling has also been emphasized [31].

#### 2.1.4 Coenzyme Q10

Coenzyme Q10 (CoQ10) is a fat-soluble antioxidant with nutritive and protective effects on DAergic neurons [32, 33]. Decreased levels of CoQ10 indicate an increased risk of PD associated with oxidative stress [34, 35]. Several studies have found that the levels of total and oxidized CoQ10 in the CSF of patients with PD are significantly higher than those of patients without neurological disorders [36, 37], nd the percentage of oxidized CoQ10 of the total CoQ10 (%CoQ10) in the CSF of the PD group was significantly higher than that of HC group [38]. Because of its potent antioxidant activity, CoQ10 can be classified as a potential substance for the treatment of PD [34].

#### 2.1.5 C-reactive protein

C-reactive protein (CRP) is an acute-phase plasma protein that is closely related to the inflammatory process [39]. Studies have reported significantly higher CRP levels in the CSF of patients with PD compared with HCs [40, 41]. Furthermore, CRP levels were significantly higher in the CSF of patients with PD with dementia (PDD) compared with patients with PD without dementia and HCs, but it was not possible to distinguish between patients with PD without dementia and HCs [42]. In conclusion, research has shown that the difference between CRP levels in the CSF in patients with PD and HCs is not significant [43, 44].

#### 2.1.6 Lysosomal protein

Lysosomes are an important link in the α-syn degradation pathway, and changes in the autophagy-lysosome system can lead to the abnormal deposition of α-syn in vivo [45]. The *GBA* gene encodes the lysosomal enzyme glucocerebrosidase (GCase); reduced levels of this enzyme cause lysosomal storage disorders [46]. Many genes associated with lysosomal storage disorders are also associated with PD susceptibility [47]. GCase activity was lower in the CSF of patients with PD carrying a *GBA* mutation (GBA-PD) compared with that in the CSF of patients with sporadic PD (sPD); moreover, GBA-PD with severe mutations showed the lowest enzyme activity, suggesting that the *GBA* mutation accelerates the pathological condition [48-50]. Patients with PD also showed impaired lysosomal function compared with HCs [51]. However, other studies have reported reduced GCase activity in the CSF of patients with PD; this reduction was independent of the *GBA* mutation and may have been due to aging [52]. The activity of other lysosomal hydrolases (e.g., β-hexosaminidase and β-galactosidase) was also reduced in the CSF of patients with GBA-PD and sPD compared with HCs [53]. In conclusion, lysosomal metabolism is closely linked to the pathophysiology of PD, and its changes can be monitored.

#### 2.1.7 Pros and cons of using biomarkers in the CSF

The CSF is attracting increasing attention as a key fluid for the potential development of biomarkers for NDDs. The main function of the CSF is to protect the CNS; in addition to its role in protecting the brain from severe shocks, it has recently been shown to carry metabolic waste out of the CNS via the glial-lymphatic system [54]. The CSF may be the most important fluid for the detection of biomarkers of CNS-associated diseases; this is mainly because it is directly connected to the CNS and can thus directly reflect physiological or pathological changes in the CNS, making the results more credible. Research has shown that t-α-syn can be detected in the CSF via enzyme-linked immunosorbent assay (ELISA, BioLegend), t-tau can be detected by a highly standardized microbead-based immunoassay (Alz Bio3 Kit, Fujirebi) [55], and DJ-1 can be detected by multi-walled carbon nanotubes (MWCNTs) and LC-MS/MS [22]. However, the clinical development of CSF biomarkers is still weak, mainly because of the trauma and contamination associated with CSF puncture. Standardized test methods for CSF marker screening are also lacking due to interindividual heterogeneity. Currently, it seems that t-tau/t-α-syn, p-α-syn, oxidized CoQ10/total CoQ10, DOPAC, and HVA are ideal CSF biomarkers. However, much research is still needed to identify and assess biomarkers from the CSF.

### 2.2 Peripheral blood

#### 2.2.1 Plasma α-syn

Conflicting data on α-syn in the plasma of patients with PD have persisted because of the diversity of research methods and sources of α-syn production [56, 57], which may be due to rupture disturbances of α-syn-containing erythrocytes [58]. Plasma levels of pS129 α-syn, t-α-syn, and o-α-syn were significantly higher in patients with PD than in HCs, as determined by ELISA [13]. By contrast, another paper reported that plasma p-α-syn alone was more sensitive and specific for diagnosing PD [59]. When comparing p-α-syn and non-p-α-syn levels longitudinally, the initial levels of p-α-syn extracted from the plasma of patients with PD were higher than the t-α-syn levels. After 20 years, the t-α-syn levels of patients increased, whereas p-α-syn levels remained unchanged [60]. In conclusion, these studies imply that p-α-syn levels can be used as a diagnostic tool, whereas t-α-syn levels can be used to evaluate disease progression.

#### 2.2.2 Serum insulin-like growth factor-1

Insulin-like growth factor-1 (IGF-1) is a polypeptide protein derived from depolarized DAergic neurons [61] with antioxidant, anti-aging, and protective effects on brain cells [62]. Serum IGF-1 expression levels were higher in patients with early PD [63] than in patients in the middle and late stages, indicating that serum IGF-1 levels are significantly negatively correlated with anxiety, depression, and cognitive impairment [63, 64]. Furthermore, the upregulation of IGF-1 expression can alleviate the symptoms of PD [65]. In summary, serum IGF-1 may be a meaningful biomarker for early PD diagnosis and the monitoring of disease progression.

#### 2.2.3 Serum lipid content

Lipid dysregulation is involved in a variety of pathological processes in PD [66] , and statins can relieve the symptoms of PD by inhibiting cholesterol synthesis in clinical practice [67]. Studies have shown that serum triglyceride (TG), total cholesterol (TC), low-density lipoprotein cholesterol (LDL-C), apolipoprotein A1 (apo A1), and apolipoprotein B (apo B) levels are significantly lower in patients with PD than in HCs [68, 69]. By contrast, patients with PD with mild cognitive impairment (PD-MCI) had higher mean levels of TC, TG, and apo A1 than subgroups with normal cognition. Therefore, TC, TG, and apo A1 could be useful biomarkers for PD-MCI [69]. Conversely, serum high-density lipoprotein cholesterol (HDL-C) levels were elevated in patients with PD [70, 71].

#### 2.2.4 Blood uric acid

Uric acid (UA) salts can resist iron-dependent forms of non-apoptotic cell death (ferroptosis) and peroxidation, which in turn can reduce damage to DAergic neurons [72, 73]. The decrease in blood UA leads to damage and necrosis in nigrostriatal DAergic neurons [74]. It is well documented that the blood UA levels of patients with PD are significantly lower than those of HCs [68] . One study reported that the blood UA levels of patients with PD with cognitive impairment was lower than those of the HC group, suggesting that UA concentration affects the incidence of PD and cognitive impairment [75]. In addition, PD can be categorized into two subtypes: tremor-dominant (TD) PD and non-tremor-dominant (NTD) PD. Patients with NTD PD have significantly lower serum UA levels and a significantly lower serum uric acid/creatinine ratio (UA/Cr) compared with patients with TD PD. Serum UA and UA/Cr were also significantly higher in patients with mild PD than in those with moderate to severe disease [76]. A higher concentration of UA results in a lower risk of PD [77]. Therefore, serum UA and UA/Cr are potentially useful biomarkers to indicate the risk, severity, and subtypes of PD.

#### 2.2.5 Malondialdehyde

Malondialdehyde (MDA) is a product of lipid peroxidation, and its levels can indirectly reflect the degree of cell damage [78]. Several oxidative stress products in the blood were detected and analyzed in patients with PD, and it was found that the blood MDA levels in patients with PD were significantly higher than those in controls. Furthermore, MDA had higher sensitivity and specificity than other markers [79], indicating severe oxidative stress injury in patients with PD [80, 81]. By contrast, in another study, serum MDA levels of patients with PD were not significantly different from those of controls. However, MDA was correlated with PD; MDA levels were negatively correlated with PD duration and positively correlated with age at onset [82].

#### 2.2.6 Plasma antioxidant enzymes

Antioxidant enzymes may reduce cellular damage due to oxidation products during PD development [83, 84]. The levels of superoxide dismutase, glutathione peroxidase, and catalase in the plasma of patients with PD were significantly lower than those in the plasma of HCs [85, 86], suggesting that the antioxidant function of patients with PD patients was significantly decreased. Moreover, plasma total glutathione levels were significantly lower in the PD-MCI group than in the normal cognition (NC) group [87]. A previous study reported that serum superoxide dismutase and catalase did not differ significantly between PD and HCs [88]. Furthermore, the levels of oxidative stress markers (thiobarbituric acid reactive substances and advanced oxidative protein products) and inflammatory markers (nucleoside triphosphate hydrolases, ischemia-modified albumin, and myeloperoxidase) were significantly elevated in the sera of patients with PD.

#### 2.2.7 Cytokine

There is growing evidence that the peripheral and adaptive immune systems are involved in the disease process of PD. α-syn also promotes the production of cytokines associated with peripheral inflammatory vesicles [89]. Inflammatory responses involving cytokines can exacerbate damage to neurons; several studies have focused on the relationship between PD and tumor necrosis factor (TNF), tumor necrosis factor receptor (TNFR), and interleukin (IL) [90, 91]. Soluble tumor necrosis factor receptor 1 (sTNFR1) is a TNF receptor that can affect neuronal development and trigger cognitive disorders [92]; a previous study revealed that the levels of serum TNF-α and sTNFR1 in patients with PD were significantly higher than those in HCs [91].

IL is closely linked to the regulation of inflammatory and immune responses [93]. Plasma samples from patients with PD had significantly higher levels of the inflammatory proteins cysteinyl asparagine-1, cysteinyl asparagine-recruiting structural domain, and IL-18 than samples from HCs [94]. In addition, the levels of other cytokines, such as TNF-α, IL-1β, IL-2, IL-4, IL-6, and interferon γ (INF-γ), in the CSF and serum of patients with PD were significantly higher than those of HCs [95, 96]. By contrast, in another study, the serum IL-6 levels of patients with PD were not higher than those of controls, while serum IL-1β, IL-2, IL-10, and TNF-α levels were significantly increased [97, 98]. However, another study reported that serum IL-6 levels did not reflect the severity of PD because they were not correlated with patients’ scores on the Unified Parkinson’s Disease Rating Scale (UPDRS) [99]. Conversely, other researchers found that IL-2 and IL-6 were associated with the progression of early PD with nonmotor symptoms (NMSs) [98]. Although the results of these studies are inconsistent, cytokine-induced inflammation may contribute to the pathological progress of PD [100].

#### 2.2.8 Chemokine

Monocyte chemotactic protein-1 (MCP-1) and macrophage inflammatory protein-1α (MIP-1α) are examples chemokines [101]. The literature reports high serum MCP-1 and MIP-1α levels in patients with PD [102], implying higher levels of inflammation in patients with PD. C-X-C motif chemokine 12 (CXCL12), belongs to the CXC subfamily and is thought to be a key contributor to neuroinflammation. CXCL12 has two receptors: C-X-C motif chemokine receptor-4 (CXCR4) and C-X-C motif chemokine receptor-7 (CXCR7) [103, 104]. CXCL12 serum levels were significantly higher in patients with PD compared with those in HCs. Moreover, CXCR4 expression in peripheral blood mononuclear cells (PBMC) was significantly increased in patients with PD compared with that in HCs [105]. Another study reported that CXCL12 expression was increased not only in blood but also in the brain tissue of patients with PD, and it was positively correlated with α-syn levels [106].

#### 2.2.9 C-reactive protein

CRP is a biomarker of systemic inflammation [107]. The pro-inflammatory factor IL-6 can induce the release of CRP [108]. Serum high-sensitivity CRP (hs-CRP) levels are elevated in patients with PD compared with HCs and vary between PD subtypes [109, 110]. In addition, plasma CRP levels in patients with PD are correlated with disease severity and motor function scores, suggesting that CRP plays a key role in the pathogenesis of PD [111]. One study reported a positive correlation between plasma hs-CRP levels and gait freezing in PD [112]. Therefore, CRP levels are a potential marker of PD.

#### 2.2.10 Homocysteine

Homocysteine (Hcy) is a sulfur-containing amino acid that can cause neurotoxicity and neuronal death [113, 114]. Many preliminary trials have demonstrated that plasma Hcy levels are higher in patients with PD than in HCs [115, 116]. Another study measured plasma Hcy levels in two PD subtypes—patients with PD without dementia (PDwoD) and patients with PDD—as well as HCs. The researchers found that patients with PDD exhibited higher Hcy levels than patients with PDwoD and HCs [117]. In addition, more advanced clinical stages of the disease were associated with higher plasma Hcy levels [118]. Overall, plasma Hcy levels were negatively correlated with cognitive function, and an elevated plasma Hcy level was a large risk factor for cognitive dysfunction in older patients with PD [119].

#### 2.2.11 Glial fibrillary acidic protein

Glial fibrillary acidic protein (GFAP) is an intermediate filament protein that is increased in reactive astrocytes, and GFAP is associated with degeneration of DAergic neurons in PD [120]. Levels of GFAP are significantly increased in the plasma of patients with PD compared with that of HCs [121, 122]. GFAP has the potential to differentiate between different subtypes of PD; plasma GFAP levels were significantly higher in patients with PD with rapid eye movement sleep behavior disorder (RBD) compared with patients the PD without RBD [123]. Moreover, plasma GFAP was elevated in patients with PDD compared with HCs, patients with PD-MCI, and patients with PD-NC. Plasma GFAP was significantly increased in patients with PD-MCI compared with HCs, demonstrating that cognitive impairment may be associated with elevated GFAP [7].

#### 2.2.12 Extracellular vesicle-related biomarkers

Extracellular vesicles (EVs) contain lipids, proteins, and nucleotides and are widely found in body fluids, such as the CSF, plasma, and urine [124]. Exosomes are a type of EV that are typically enriched in non-coding RNA (ncRNA). To avoid the interference of α-syn in erythrocytes, several methods can quantify α-syn in neuron-derived exosomes in the blood [125, 126]. In different cohorts of patients with PD, α-syn in serum exosomes was consistently elevated [127]. The plasma exosomal α-syn/t-α-syn (exosomes/total) ratio was also significantly higher in patients with PD than in controls. In addition, plasma exosomal α-syn was positively correlated with disease severity [96].

A comparison of plasma microRNA (miRNA) between patients with early-onset PD, patients with late-onset PD, and HCs detected a statistically significant difference between patients with PD and HCs. Among these miRNAs, upregulated miR-29b-3p and downregulated miR-297, miR-4462, etc., may be associated with early-onset PD only [128]. Furthermore, patients with PD could also be distinguished from HCs by the combined detection of miR-133b, miR-221-3p, and miR-4454 [129]. In addition, plasma exosomes exhibit dopamine neuronal protective ability and brain targeting ability [130]; therefore, they may also be important for the treatment of PD.

#### 2.2.13 Pros and cons of using biomarkers in the peripheral blood

Blood sample collection is common in clinical practice; it is less invasive than CSF collection and facilitates population screening [131]. However, markers in the blood are often not as accurate as markers in the CSF. Furthermore, the composition of blood is more complex than that of the CSF; blood reflects changes in the whole organism rather than just the CNS. Therefore, identifying PD with blood markers must account for the presence or absence of other pathologies that may cause increased or decreased levels of the marker. In patients with early PD, plasma levels of IGF-1 are higher than those in patients with late-stage PD, indicating that IGF-1 could enable the early diagnosis of PD. However, IGF-1 levels decrease with age, so IGF-1’s level of diagnosis and normal physiological levels must be identified. Moreover, although a decrease in IGF-1 may indicate the progression of PD, it may be also associated with other diseases [62]. Blood UA in patients with PD is lower than that in healthy individuals and negatively correlates with disease severity, and UA/Cr can differentiate between tremor-dominant and non-tremor-dominant PD. Therefore, this marker may also be a prognostic tool, predicting the risk of developing PD as well as its severity. Evidence indicates that the levels of lipid such as plasma TC, TG, apo B, apo A1, and HDL-C can be used to monitor disease progression and risk factors; however, lipid regulation may also represent a future treatment target for psychiatric disorders related to PD, such as anxiety disorders and cognitive disorders. Hcy levels in patients with PD are negatively correlated with the level of cognitive performance. The detection of MDA in the blood of patients with PD is still controversial and must be differentiated from aging-related indicators. The cytokines TNF-α, sTNFR1, IL, and INF-γ in the serum of patients with PD can be used to monitor the progression of the disease, but the use of IL-6 as a PD marker is still controversial. In recent years, the presence of α-syn and ncRNA in EVs has become a hot research topic. Moreover, plasma exosomes possess a protective effect on neurons; therefore, they may be useful for treatment development.

### 2.3 Saliva

#### 2.3.1 Caffeine

Caffeine (CAF) can protect nerve cells by decreasing neuroinflammation [132, 133]. CAF may be utilized to determine the pathological course of PD because basal CAF levels are lower in patients with mid- to late-stage PD than in those with early- and new-onset PD [134]. In addition, lower basal CAF levels were associated with higher disease severity and duration, and basal salivary CAF levels were significantly lower in patients with PD with motor complications than in those without motor complications [134].

#### 2.3.2 α-Syn

α-Syn can be detected in saliva and has gradually become a hot research topic because saliva collection is non-invasive and easy [135]. Moreover, it can be used to differentiate PD from other NDDs [136]. Compared with HCs, salivary o-α-syn levels were significantly higher in patients with PD, and the o-α-syn/t-α-syn ratio was also higher; by contrast, p-α-syn/t-α-syn and p-α-syn/o-α-syn were lower in patients with PD than in HCs [137, 138]. Meanwhile, t-α-syn detection via RT-QuIC was able to differentiate between MSA and PD [139]. Single t-α-syn, o-α-syn, and p-α-syn assay results revealed poor sensitivity, but the combination of the p-α-syn/t-α-syn ratio and o-α-syn achieved high sensitivity and specificity (80% and 78%, respectively) [140]. However, to improve the specificity of protein detection, standardized procedures and analysis are still needed [141].

#### 2.3.3 Pros and cons of using biomarkers in saliva

The advantages of saliva samples are that they have large volumes and are rich in substances [142], thus, they can be applied not only in the diagnosis and detection of PD but also in other NDDs, such as AD and MSA. Proteins, nucleic acids, and antioxidants have been increasingly studied as potential biomarkers of PD saliva. Although single markers lack sufficient specificity and sensitivity, α-syn-related markers can distinguish patients with PD from healthy individuals and patients with other NDDs. In addition, CAF can be used as a tool for disease monitoring.

### 2.4 Urine

#### 2.4.1 Amino acids

Urine contains abundant metabolites, including various amino acids [143]. A previous study reported that patients with PD had significantly increased concentrations of ornithine, phenylalanine, isoleucine, β-hydroxybutyric acid, tyrosine, and succinic acid compared with HCs. These metabolites are associated with multiple impaired metabolic pathways in patients with PD, including ornithine and the tricarboxylic acid cycle [144]. Therefore, they may indicate the severity of the disease. For example, the concentration of succinic acid was positively correlated with exercise scores. Furthermore, increased excretion of valine, isoleucine, and leucine is associated with PD staging [145], indicating that urine amino acids are feasible diagnostic markers for PD.

#### 2.4.2 8-OHdG

8-OHdG is the main product of oxidative DNA damage and is excreted in the urine [146, 147]. It was demonstrated that 8-OHdG measured alone and the ratio of 8-OHdG/2-dG (the corresponding non-hydroxylated base 2'-deoxyguanosine) in the urine and plasma is significantly higher in PD patients than in HCs [148], and the mean urinary 8-OHdG increases with increasing stages of PD [149], suggesting that this marker can be used to monitor disease progression. Conversely, 8-OHdG has been reported to be elevated in PD with hallucinations but not in PD with dementia or other clinical features [150, 151]; thus, it may also be used for PD staging.

#### 2.4.3 Bis(monoacylglycero)phosphate

A proportion of patients with PD contain mutations in the leucine-rich repeat kinase 2 (*LRRK2*) gene. The bis(monoacylglycerol)phosphate (BMP) isoform is a detectable product of this mutation and can be found in urinary exosomes. *LRRK2* carriers with PD have higher levels of BMP than carriers without PD, and BMP levels can be associated with a decline in cognitive levels of patients with PD [152]; thus, BMP could be used to monitor the progression of PD.

#### 2.4.4 Pros and cons of using biomarkers in urine

Urine is an ideal source of biomarkers because it can be easily and noninvasively collected and is sample-rich, containing nucleic acids, proteins, lipids, and even biomarkers of distal brain origin. However, because of the low concentration of markers and confounding variables (personal circumstances, diet, medications, etc.), no clear and valid key markers have been identified. As mentioned above, mean urinary amino acids, 8-OHdG, and exosomal BMP can be used in the diagnosis, staging, and progression monitoring of PD, and urinary ISA can be used to differentiate patients with PD from healthy individuals. Other noteworthy markers for diagnosing early PD include urinary kynurenine (KYN) [153], which has high specificity (90.2%) and positively correlates with PD severity and mild cognitive impairment; it also has potential for diagnosing and monitoring disease progression. By contrast, the diagnostic value of modified nucleosides [154] and oxidized DJ-1 (OxiDJ-1) [155] for detecting early PD is still debated. Currently, diagnosing PD on the basis of biomarkers in the urine is not a common method. The identification of a urinary biomarker that could be used to diagnose PD with high sensitivity and specificity would be a major step forward. Because urine sample collection is more convenient and less painful for the patient than the collection of other samples, using biomarkers in the urine could also be used as a routine indicator in medical examinations or for self-monitoring. Therefore, these biomarkers could be used for the early diagnosis and treatment of PD and thus have a wide range of applications.

## 3. Biomarkers in tissues

### 3.1 Brain iron

In the brain, increased iron levels may lead to oxidative cell damage and neuroinflammation [156]. In addition, iron deposition can lead to increased expression and aggregation of α-syn [157, 158]. Some experiments have reported higher iron content in the SN in post-mortem brain tissue from patients with PD compared with HCs [159, 160]. Results detected by quantitative susceptibility mapping (QSM) showed that the magnetic susceptibility of patients with PD was substantially higher, especially in patients with severe symptoms [161]; thus, PD progression could be detected by measuring iron deposition in the brain [162, 163]. Furthermore, different symptoms could be predicted on the basis of increased iron levels in different brain regions [164].

### 3.2 Gut biomarkers

Patients with PD typically have intestinal dysfunction (e.g., constipation), and the gut flora can affect the physiological activities of the brain [165, 166]. Patients with PD have significantly lower levels of lactic acid bacteria and enterococci, whereas *Bifidobacterium* and *Enterobacteriaceae* are more abundant in these patients [167, 168]. Moreover, the gut flora influences the progression of PD [169, 170]. Additionally, early PD is associated with a reduction in bacterial metabolites, namely short-chain fatty acids (SCFAs), as well as an inflammatory response in the intestines; this association can be used to help diagnose PD [171]. In addition, several studies have found that patients with PD have higher levels of various immune factors, namely FMS-associated receptor tyrosine kinase 1 (Flt1), IL-1α and CXCL8 [171], and calcineurin [172]. However, the efficacy of intestinal markers can be affected by many confounding variables, and the related results are highly skewed [173]. Therefore, the selection of intestinal markers requires error correction that must consider the complex effects of metabolomics, dietary and pharmacological datasets, and microbiota.

### 3.3 Skin p-α-syn

p-α-Syn and α-syn can be detected in the nerve fibers of the skin in patients with PD [174]. Some studies have shown increased p-α-syn content in the skin of patients with PD [175, 176]. Other researchers demonstrated that p-α-syn could be observed in cutaneous nerve fibers and the spine in patients with idiopathic PD (IPD) but not HCs [177, 178]. Therefore, p-α-syn could be used as a potential biochemical marker for the diagnosis of IPD.

## 4. Detection methods and kits for clinical application

### 4.1 Clinical diagnostic methods

Currently, the clinical diagnosis and staging of PD depend mainly on motor characteristics and imaging modalities, including molecular imaging, transcranial ultrasound, magnetic resonance imaging (MRI), and optical coherence tomography (OCT). However, the clinical symptoms of PD often overlap with those of other NDDs, and the early motor symptoms of PD are hidden, so accurate diagnosis of PD is difficult. Therefore, the identification of a set of sensitive and specific biomarkers is particularly important for the early diagnosis, timely intervention, and disease monitoring of PD. As a result, kits for these biomarkers have become a hot research topic in the field of PD diagnosis.

#### 4.1.1 Genetic test

According to genome-wide association studies (GWASs) on PD, genetic variants contribute to approximately 16-36% of the risk of PD [179]. Familial PD is associated with rare high-prevalence single-gene variants [180] in the α-syn gene (*SNCA*), *VPS35*, *PARK7*, *PINK1*, and *PRKN*, whereas low-prevalence genetic variants are often associated with sporadic PD. Distinguishing between high- and low-prevalence variants is likely to be useful in predicting sporadic or familial PD. Furthermore, other common genetic variants increase the risk of PD, such as *LRRK2* and glucocerebrosidase (*GBA*) variants [181]. *SNCA* duplications are pathogenic and increase the expression of α-syn, which can exacerbate the symptoms of the disease [182], and *SNCA* duplication triplets are associated with early-onset disease and cognitive impairment, suggesting a gene dosage effect. *PRKN* heterozygotes account for half of early-onset PD cases [180]. Moreover, a GWAS demonstrated that sporadic PD was correlated with *SNCA*, *RAB29*, *MAPT*, *BST1*, *GAK*, *LRRK2*, and *HLA-DRB5* [183].

**Table 1:** Parkinson's disease detection kit.

Name	Detection object	Applicant	References
**Combined marker and detection kit for the diagnosis of Parkinson’s disease**	Plasma small molecule metabolites caffeine, creatinine, eicosanamide, phenylacetylglutamine, capric acid and indole lactic acid	First Affiliated Hospital of DaLian Medical University	[197]
**Gene diagnosis kit for Parkinson's disease**	SNCA, Parkin, Pink1, UCHL-1, DJ-1, ATP13A2, GIGYF2, HTRA2, FBX07, Vps35, MAPT	JiangSu XiongMing Pharmaceutical co.ltd.	[198]
**THBD gene as a molecular marker for the diagnosis of Parkinson's disease**	The expression of THBD gene	QingDao YangShen Bio-medicine co.ltd.	[185]
**Kit for detecting Parkinson's syndrome**	The expression of angiopoietin-like protein 4	Jiangsu Nuo Ming Zhe Tian Medicial Inspection Laboratory co.ltd.	[199]
**A diagnostic kit for detecting Parkinson's disease**	Brand-new pathogenic gene mutation site	West China Hospital SiChuan University	[200]
**Reagent kit and device for early detection and diagnosis of Parkinson's disease**	The expression of CHCHD2 gene in erythrocyte	Peking University	[201]
**Kit for diagnosing Parkinson’s disease and method for diagnosing Parkinson’s disease**	Mydriasis volume of eyedrops	Kansai Tlokk	[202]
**Kit for diagnosis of Parkinson’s disease**	Autoantibody level of ATG4B protein	Yep Bio co.ltd.	[200]
**Method for diagnosing Parkinson’s disease using nasal mucus, composition therefore, and kit comprising the same**	α-syn (SNCA), parkin, ZNF746 (PARIS), RNF146, c-Abl and AIMP2	Research & Business Found Sungkyunk Wan Univ; IUCF HYU	[203]

MAPT: Microtubule-Associated Protein Tau; Vps35: vacuolar protein sorting 35 homolog; FBX07: F-box protein 7; HTRA2: high-temperature requirement serine protease A2; GIGYF2: GRB10 Interacting GYF Protein 2; ATP13A2: Probable cation-transporting ATPase 13A2; UCHL-1: Recombinant Ubiquitin Carboxyl Terminal Hydrolase L1; PINK1: PTEN-induced kinase 1SNCA: Synuclein alpha Gene; THBD: thrombomodulin; CHCHD2: Coiled-Coil-Helix-Coiled-Coil-Helix Domain Containing 2; ATG4B: Autophagy related 4B Cysteine Peptidase; ZNF746: Zinc Finger Protein 746; RNF146: Ring Finger protein 146; c-Abl: Non-receptor tyrosine kinases; AIMP2: Aminoacyl-tRNA synthetase complex interacting multifunctional protein-2.

The clinical use of genes and gene mutation expression products to detect PD is very common, and some kits can detect mutations in *POLG1*, the gene encoding mitochondrial DNA polymerase, in patients with hereditary PD [184]. Previous research detected the *THBD* gene in the blood to diagnose and estimate the risk of PD [185]. The diagnosis of PD can also be confirmed by detecting the expression of the *CHCHD2* gene in erythrocytes [186]. In addition, a study used skin sampling to diagnose PD by measuring the levels of at least one of the following genes or its expression product: *SNORA16A*, *SNORA24*, *SNORA50*, and *REXO1L2P* [187]. Another study evaluated PD according to *GRK5* expression and screened for PD therapeutics [188]. In addition, researchers developed a kit to detect 11 PD-linked genes, namely *SNCA*, *PRKN*, *PINK1*, *UCHL-1*, *DJ-1*, *ATP13A2*, *GIGYF2*, *HTRA2*, *FBX07*, *VPS35*, and *MAPT* [189].

#### 4.1.2 Antibody, protein detection

Early PD diagnosis is possible with the antigen-antibody specific binding method, which involves examining the binding of known antigens to specific antibody markers in a patient’s blood, such as autoantibodies that specifically bind to serine/threonine-protein kinase MARK1, tRNA pseudouridine synthase-like 1 (PUSL1), IL20, and C-C motif chemokine 19 (CCL19) [190, 191]. Other kits contain antibodies that specifically bind to phosphorylated Ser65 of Parkin and Thr257 of PINK1 [192]. This method can also be used to detect changes in the contents of specific proteins, such as CD38 and α-syn, in the patient’s serum or plasma [193]. Xu et al [194] used antigen-antibody binding to detect the level of lymphocyte activation gene-3 (LAG-3) in the blood, CSF, and other body fluids of patients, reflecting the deposition of α-syn in these body fluids. In addition, ELISA kits can be used to determine GFAP levels in the CSF and 8-OHDG and DJ-1 levels in the plasma.

**Table 2:** The content of biomarkers in PD patients.

Position	Biomarkers		Content in PD
**CSF**	α-syn	t-α-syn	Decrease
		o-α-syn	Increase
		p-α-syn	
	DA metabolites		Decrease
	DJ-1		
	Co Q10		Increase
	CRP		
	Lysosomal protein	Gcase	Decrease
**Peripheral Blood**	α-syn	t-α-syn	Increase
		o-α-syn	
		p-α-syn	
	IGF-1		
	Lipid content	Apo A1	Decrease
		Apo B	
		TG	
		TC	
		LDL-C	
		HDL-C	Increase
	UA		Decrease
	MDA		Increase
	Antioxidant enzymes		Decrease
	Cytokine	TNF-α	Increase
		sTNFR1	
		IL-1β	
		IL-2	
		IL-10	
		IL-6	Remains to be discussed
	Chemokine	CXCL12	Increase
		CXCR4	
	CRP		
	Hcy		
	EV related biomarkers	α-syn	
		miR-29b-3p	
		miR-297	Decrease
		miR-4462	
**Saliva**	CAF		Decrease with process
	α-syn	t-α-syn	Decreased or unchanged
		o-α-syn	Increase
**Urine**	Amino acids		Increase
	8-OHdG		Increase with process
	EV related biomarkers	BMP	No difference from HC
**Tissue**	Brain iron		Increase
	Gut	Flt1	
		CXCL8	
		IL-1α	
	skin p-α-syn		

CSF: cerebrospinal fluid; α-syn: α-synuclein; t-α-syn: total α-synuclein; o-α-syn: oligomeric α-synuclein; p-α-syn: phosphorylated α-synuclein; DA: dopamine; Co Q10: Coenzyme Q10; CRP: C-reactive protein; Gcase: glucocerebrosidase; IGF-1: insulin-like growth factor-1; Apo A1: apolipoprotein A1; Apo B: apolipoprotein B; TG: triglyceride; TC: total cholesterol; LDL-C: low-density lipoprotein cholesterol; HDL-C: high-density lipoprotein cholesterol; UA: uric acid; MDA: malondialdehyde; TNF-α: tumor necrosis factor-α; sTNFR1: soluble tumor necrosis factor receptor 1; IL-1β: interleukin-1β; IL-2: interleukin-2; IL-10: interleukin-10; IL-6: interleukin-6; CXCL12: C-X-C motif chemokine 12; CXCR4: C-X-C motif chemokine receptor-4; Hcy: homocysteine; EV: extracellular vesicles; miR: microRNA; CAF: caffeine; 8-OHdG: 8-hydroxy-2'-deoxyguanosine; BMP: bis (monoacylglycerol) phosphate; Flt1: FMS-associated receptor tyrosine kinase 1; CXCL8: C-X-C motif chemokine 8; IL-1α: interleukin-1α.

#### 4.1.3 Alternative methods

Because the expression levels of some miRNAs are increased or decreased in patients with PD, nucleic acid hybridization can be utilized to determine miRNA contents and thereby diagnose PD [195]. One study selected miRNA molecules or their precursors in the peripheral blood plasma as detection indicators and identified disease-related genes through the miRNA contents to determine potential therapeutic targets [83]. In addition, detecting changes in specific microbial populations in the feces of PD patients can be used to help diagnose PD [196].

### 4.2 Clinically approved kits

Most kits used to diagnose PD use the CSF, blood, saliva, and urine as test samples to detect the causative genes of PD and their downstream metabolites and biomarkers (including gene mutations or polymorphisms, gene expression products, peptides, proteins, lipid metabolites, and other small molecules) (Table 1). These kits have the advantages of convenient and rapid detection, low detection cost, and easy accessibility; furthermore, they can be used to screen high-risk groups and have very wide application prospects. However, these tests also have several major drawbacks. Because of the relatively low concentration of biomarkers in these body fluids, differences in the test findings are frequently nonsignificant, which may indicate low sensitivity. Despite these limitations, kit detection is unquestionably the best option for PD diagnosis, and this method promises to become more convenient and popular in the future. Therefore, it is urgent to identify biomarkers and develop detection methods with high sensitivity and specificity.

## 5. Perspective on biomarkers for early diagnosis of PD and therapeutics

Over the past decade, among the many biomarkers related to PD, α-syn and its related molecules have gradually become the focus for early PD detection. As a clinical biochemical marker in early PD, the specificity, sensitivity, and repeatability of the biomarker analysis must fall within an acceptable range, and differences between individuals must be minimized. Furthermore, these biomarkers must have high stability to facilitate sample preservation. Previous studies have found that many biomarkers have inconsistent test results due to different types of PD, different disease processes, and the gender and age of the patients. Because relying on a single biochemical marker leads to a high misdiagnosis rate, the combination of α-syn with other biomarkers may yield better outcomes for early PD diagnosis. We list PD-related biomarkers that have changed in level during the course of the disease (Table 2).

## 6. Conclusions

Currently, CSF analysis provides a generally stable set of tests for the diagnosis of brain disorders, but CSF collection is invasive. Fortunately, other easily accessible samples (peripheral blood, saliva, urine, skin, etc.) contain many promising biomarkers. The collection of these samples is minimally invasive, nonintrusive, more easily accepted by patients, and simple to popularize and promote.

As more and more specific markers of PD are discovered, PD detection is becoming more accurate and convenient, and different PD subtypes (hereditary PD, secondary PD, etc.) can be diagnosed more clearly. This also means that combined assays will have great scope for development and good application prospects. Although the current assays and detection methods still have many limitations, these differences caused by different detection methods provide a wide variety of models for combined assays and studies. This joint detection includes the detection and judgment of various markers in different body fluids and other samples, and it aligns with many definite factors for the diagnosis of PD in the future. The choice of which models (disease duration, region, age, etc.) and tests should be used in clinical practice will be an important theme in the early clinical diagnosis of PD in the future.
